# Impact of statins and ACE inhibitors on mortality after COPD exacerbations

**DOI:** 10.1186/1465-9921-10-45

**Published:** 2009-06-03

**Authors:** Eric M Mortensen, Laurel A Copeland, Mary Jo V Pugh, Marcos I Restrepo, Rosa Malo de Molina, Brandy Nakashima, Antonio Anzueto

**Affiliations:** 1 VERDICT research unit, South Texas Veterans Health Care System, San Antonio, Texas, USA; 2 Department of Medicine, Division of General Internal Medicine, University of Texas Health Science Center at San Antonio, San Antonio, Texas, USA; 3 Department of Psychiatry, University of Texas Health Science Center at San Antonio, San Antonio, Texas, USA; 4 Department of Epidemiology and Biostatistics, University of Texas Health Science Center at San Antonio, San Antonio, Texas, USA; 5 Deparment of Medicine, Division of Pulmonary and Critical Care Medicine, University of Texas Health Science Center at San Antonio, San Antonio, Texas, USA

## Abstract

**Background:**

The purpose of our study was to examine the association of prior outpatient use of statins and angiotensin converting enzyme (ACE) inhibitors on mortality for subjects ≥ 65 years of age hospitalized with acute COPD exacerbations.

**Methods:**

We conducted a retrospective national cohort study using Veterans Affairs administrative data including subjects ≥65 years of age hospitalized with a COPD exacerbation. Our primary analysis was a multilevel model with the dependent variable of 90-day mortality and hospital as a random effect, controlling for preexisting comorbid conditions, demographics, and other medications prescribed.

**Results:**

We identified 11,212 subjects with a mean age of 74.0 years, 98% were male, and 12.4% of subjects died within 90-days of hospital presentation. In this cohort, 20.3% of subjects were using statins, 32.0% were using ACE inhibitors or angiotensin II receptor blockers (ARB). After adjusting for potential confounders, current statin use (odds ratio 0.51, 95% confidence interval 0.40–0.64) and ACE inhibitor/ARB use (0.55, 0.46–0.66) were significantly associated with decreased 90-day mortality.

**Conclusion:**

Use of statins and ACE inhibitors prior to admission is associated with decreased mortality in subjects hospitalized with a COPD exacerbation. Randomized controlled trials are needed to examine whether the use of these medications are protective for those patients with COPD exacerbations.

## Introduction

In the United States chronic obstructive pulmonary disease (COPD) is the 4^th ^leading cause of death overall [1], and is frequently complicated by recurrent acute exacerbations, which are nationally responsible for 110,000 deaths and >500,000 hospitalizations per year [2]. Although extensive research on therapies has been conducted to improve survival for patients with COPD so far only oxygen therapy, lung volume reduction surgery, and smoking cessation have been definitively demonstrated to improve survival for patients with COPD [3].

In COPD, airway pro-inflammatory cytokine levels have been demonstrated to be associated with increased airway obstruction and exaggerated airway inflammatory response [4,5]. In addition, elevated levels of pro-inflammatory cytokines, including IL-8 and TNF-alpha, have been associated with increased incidence of respiratory infections [6] and worse clinical outcomes including increased mortality and poor health status [7-10]. Several studies have demonstrated that HMG-CoA reductase inhibitors ("statins") and angiotensin converting enzyme (ACE) inhibitors have significant immunomodulatory effects and reduce systemic cytokine levels [11-15]. There have been several recent pharmacoepidemiologic studies that have demonstrated that statin [16-19] and/or ACE inhibitor use [17] were associated with improved outcomes for patients hospitalized with acute COPD exacerbations or for those with pre-existing COPD. However these studies all had important limitations including small sample sizes from single sites, incomplete risk adjustment, or not using techniques to minimize immortal time bias. Therefore further research is needed to clarify the roles and importance of these medications in the treatment of patients with acute exacerbation of COPD.

The study aims were to assess the association of the use of statins and ACE inhibitors on mortality in a population of largely male subjects ≥ 65 years of age hospitalized with acute COPD exacerbations after adjusting for other potential confounders using the extensive administrative databases of the Department of Veterans Affairs (VA).

## Methods

This study was conducted with VA inpatient and outpatient administrative data that was collected as part of a larger study of inappropriate prescribing practices in the elderly [20]. The Institutional Review Board of the University of Texas Health Science Center at San Antonio classified this as an exempt study.

### Inclusion and Exclusion Criteria

Subjects who were: a) aged 65 and older on October 1 1999, b) had at least one outpatient clinic visit during fiscal year (FY) 1999 (October 1 1998 – September 30 1999), c) were hospitalized during FY 2000 with a primary discharge diagnosis of acute exacerbation of COPD (International Classification of Disease-9 codes 490–492.8, 494, 496), d) and received at least one of the following respiratory medication(s) within 90-days of presentation (e.g. any form of β-agonist, inhaled corticosteroid, tiotroprium, or ipratropium.) We excluded subjects with a history of asthma. If a subject was admitted more than once during the study period, only the first hospitalization was included. Unfortunately pulmonary function test data was not available as part of these databases to confirm the diagnosis of COPD. However a recent publication by Joo et al., using a similar methodology to define COPD demonstrated that >90% of subjects were Global Initiative on Chronic Obstructive Lung Disease class 3–4 [21].

### Data

This study used data from the National Patient Care Database at the Austin Automation Center, pharmacy data from the VA Pharmacy Benefit Management group, and vital status data from the Beneficiary Identification Records Locator Subsystem death file and inpatient portion of the National Patient Care Database. Encrypted patient identifiers were used to link the information from each database for each subject.

Demographic information (age, sex, race, marital status, socioeconomic status) was obtained from inpatient and outpatient data. Missing race data were supplemented using self-reported race from the 1999 Large Health Survey of Veterans, a nationally representative survey of VA enrollees (July 1, 1999 – January 1, 2000) [22]. Race categories included white, black, Hispanic, and other/unknown. In addition, we utilized information on the VA means test as a surrogate for income.

Comorbid conditions were obtained from inpatient and outpatient administrative data. Charlson's comorbidity score was used to assign a comorbidity score for preexisting comorbid conditions [23,24]. Charlson's comorbidity score is based on 19 comorbid conditions each of which has an associated prognostic weight ranging from 1 to 6. Age was not included in the Charlson's score and was evaluated separately.

Pharmacy data were obtained from the Pharmacy Benefits Management group databases. Subjects were considered a "current user" of a given medication if their last filled prescription included enough pills to last until the date of hospitalization assuming an 80% compliance rate, which is based on prior research of medication compliance [25,26]. Medications classified as statins were atorvastatin, cerivastatin, fluvastatin, lovastatin, pravastatin, and simvastatin. Medications classified as ACE inhibitors were benazepril, captopril, enalapril, fosinopril, lisinopril, moexipril, quinapril, and ramipril. In addition, we included angiotensin II receptor blockers (ARBs) in the ACE inhibitor category for the analysis due to the small number of subjects using this class of medications. ARBs included candesartan, irbesartan, losartan, telmisartan, and valsartan.

To further control for potential confounding by medications, a count of unique drugs in each of the following classes per patient was calculated for drugs refilled/filled within 90-days of presentation: cardiac medications (excluding statins, ACE inhibitors, ARBS, and non-statin lipid lowering agents), diabetic medications, and respiratory medications. Respiratory medications included short-acting bronchodilators (albuterol, terbutaline, ipratropium), long-acting inhaled bronchodilators (salmeterol, formoterol and tiotropium), and theophylline. Two dichotomized variables were created for corticosteroid use: one for inhaled steroids and another for oral steroids. Previous research has demonstrated that using the count of these medication classes is preferable to adjusting for the individual medications [27].

In addition, we created a category of non-statin lipid lowering agents (e.g. niacin, bile acid sequestrants, and fibric acid derivatives) filled within 90-days of hospital presentation so as to examine confounding in our models.

### Outcome

Our primary outcome, 90-day mortality, was assessed using the Beneficiary Identification Records Locator Subsystem and the National Patient Care Database. Previous studies have demonstrated that after 1972 this methodology had a sensitivity of ~96% for veterans' deaths [28].

### Statistical Analyses

Bivariate statistics were used to test the association of sociodemographic and clinical characteristics with all-cause 90-day mortality. Categorical variables were analyzed using the Chi-square test and continuous variables were analyzed using Student's t-test. Due to the number of statistical tests and the sample size of this database we set statistical significance at p ≤ 0.001.

A propensity score technique was used to balance covariates associated with medication use between groups [29-31]. We created separate propensity scores for statins, ACE inhibitors/ARBs, and to examine potential confounding, non-statin lipid lowering medications. The propensity score was derived from a logistic regression model. We included variables in the propensity score if previous research demonstrated a relationship between a variable and COPD-related mortality, or if we hypothesized that it may be related to prescription of the medications (e.g. geriatric clinic use). Classes of medications included cardiac medications (excluding ACE inhibitors, ARBs, statins, and non-statin lipid lowering medications), diabetic medications, respiratory medications, inhaled corticosteroids, and oral corticosteroids. The covariates included in the propensity score models were age, gender, race, marital status, socioeconomic status, classes of medications, and the Charlson composite score. We then created an ordered categorical variable based on a quintile stratification of the propensity score to include in the regression models.

To analyze time-to-death for subjects by medication use (statin or ACE inhibitor/ARB) we used Cox proportional hazard models to estimate, and graph, the baseline survivor functions after adjusting for the respective propensity score.

Our primary analysis employed generalized linear mixed-effect models with the patient's hospital as a random effect and 90-day mortality as the dependent variable. We created separate models with either ACE inhibitors or statin use as the independent variables of interest, and adjusting for the appropriate propensity score. In addition, as secondary analyses we created similar models with the dependent variable of 30-day mortality.

We used a similar methodology to examine the association of non-statin lipid lowering drugs with 90-day mortality after adjusting for potential confounders using the appropriate propensity scores. We hypothesized that non-statin lipid lowering medications would not be associated with mortality since they have not been demonstrated to have immunomodulatory effects.

In addition, to explore whether these associations with mortality were only for those with pre-existing vascular disease we repeated our analyses stratifying by the presence of specific comorbid conditions (e.g. myocardial infarction, congestive heart failure, stroke, and diabetes).

All analyses were performed using STATA 9 (College Station, Texas).

## Results

Of the patients who had an inpatient stay in 2000 (n = 142,169), 11,212 subjects (7.9%) met our inclusion criteria. The mean age was 74.0 years with a standard deviation (SD) of 5.6 years, 42.0% of subjects were married, and 98.0% were male. In this cohort 12.8% were black, 82.3% were white, 2.7% were Hispanic, and 2.2% were other/unknown. In our cohort 6.5% of the subjects died within 30-days of presentation, and 12.4% died within 90-days of presentation. Table 1 shows demographic and comorbid condition relationships with use of statins, ACE inhibitors, and/or ARBs. Factors significantly associated with receipt of the medications of interest included lower age, not being married; history of myocardial infarction, congestive heart failure, peripheral vascular disease, stroke, diabetes with or without complications, and renal disease; and not having a history of dementia. Table 2 shows the variables examined and their relationship with 90-day mortality. The only demographic variable associated with 90-day mortality in the bivariate analysis was increasing age. Comorbid conditions associated with increased mortality included congestive heart failure, renal disease, malignancy, and metastatic solid tumors.

**Table 1:** Characteristics of subjects (N = 11,212) with acute exacerbation of COPD by use of statin and/or ACE inhibitors versus non-users of either medication*

**Variables**	**Users of statins or ACE inhibitors** **(N = 4711)**	**Non-users** **(N = 6501)**	**P-value**
Age (mean, SD)	73.5 (5.4)	74.3 (5.8)	<0.0001
Men	4616 (98)	6377 (98)	0.7
Race			
White	3829 (81)	5397 (83)	
Black	653 (45)	785 (54)	
Hispanic	124 (3)	176 (3)	0.04
Married	**2219 (47)**	**2492 (39)**	<0.0001
**Charlson Comorbid Conditions**			
Myocardial infarction	956 (20)	568 (9)	<0.0001
Congestive heart failure	2565 (54)	2001 (30)	<0.0001
Peripheral vascular disease	881 (19)	880 (14)	<0.0001
Stroke	867 (18)	819 (13)	<0.0001
Peptic ulcer	368 (8)	493 (8)	0.7
Rheumatologic disease	125 (3)	158 (2)	0.5
Mild liver disease	40 (0.8)	82 (1)	0.04
Dementia	156 (3)	324 (5)	<0.0001
Diabetes	1678 (36)	1324 (20)	<0.0001
Diabetes with complications	488 (10)	258 (4)	<0.0001
Moderate Liver disease	19 (0.4)	36 (0.6)	0.3
Hemiplegia	72 (2)	70 (1)	0.04
Renal disease	480 (10)	393 (6)	<0.0001
Any malignancy	913 (19)	1373 (21)	0.02
Metastatic solid tumor	134 (3)	245 (4)	0.008

*Data are presented as number (%) or mean (standard deviation)

**Table 2:** Characteristics of subjects (N = 11,212) with COPD exacerbation by vital status at 90-days after admission*

**Variables**	**Alive Within 90 days** (N = 9825)	**Dead within 90-days** (N = 1387)	**P-value**
Age, years mean (SD)	73.8 (5.6)	75.0 (5.8)	<0.0001
Men	9627 (98)	1366 (98)	0.2
Race/ethnicity			
White	8059 (82)	1167 (84)	
Black	1276 (13)	162 (12)	
Hispanic	269 (3)	31 (2)	0.1
Other/unknown	221 (2)	27 (2)	
Married	4788 (49)	687 (50)	0.3
**Comorbid conditions**			
Myocardial infarction	1335 (14)	189 (14)	0.97
Congestive heart failure	4800 (49)	741 (53)	0.001
Peripheral vascular disease	2703 (28)	404 (29)	0.2
Stroke	1012 (10)	153 (11)	0.4
Peptic ulcer	1411 (14)	173 (12)	0.06
Rheumatologic disease	511 (5)	62 (5)	0.2
Diabetes	3020 (31)	386 (28)	0.03
Diabetes with complications	1322 (13)	153 (11)	0.01
Liver disease, mild	291 (3)	56 (4)	0.03
Liver disease, moderate	41(0.4)	14 (1)	0.003
Hemiplegia	375 (4)	57 (4)	0.6
Dementia	415 (4)	65 (5)	0.4
Renal disease	943 (10)	180 (13)	<0.0001
Any malignancy	3068 (31)	500 (36)	<0.0001
Metastatic solid tumor	410 (4)	123 (9)	<0.0001
**Medications**			
ACE Inhibitor/ARB	3249 (33)	335 (24)	<0.0001
Statin	2113 (22)	173 (13)	<0.0001
Cardiac, count (SD)	4.8 (4.0)	4.7 (4.0)	0.7
Respiratory, count (SD)	4.4 (3.7)	4.4 (3.7)	0.7
Diabetic, count (SD)	3.0 (2.2)	3.0 (2.2)	0.5
Inhaled corticosteroids	2667 (27)	394 (28)	0.3
Oral corticosteroids	2650 (27)	390 (28)	0.4

*Data are presented as number (%) or mean (standard deviation).

In our cohort 20.4% (n = 2286) were on statins, 30.4% (n = 3404) were on ACE inhibitors, and 1.6% (n = 180) were on ARBs. Due to the small number of subjects using ARBs, for all analyses ACE inhibitor and ARB use was combined into one variable. In the bivariate analyses, both statin and ACE inhibitor use was significantly associated with decreased 90-day mortality. No other medication classes were significantly associated with mortality in our bivariate analyses.

**Figure 1:**
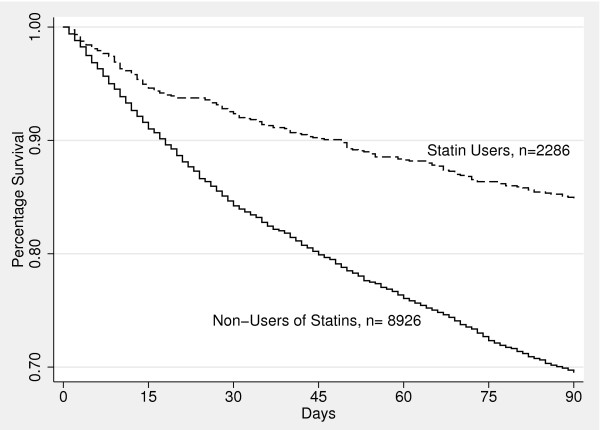
**Proportion of surviving patients hospitalized with COPD exacerbation by use of statin versus non-use (p < 0.0001)**.

**Figure 2:**
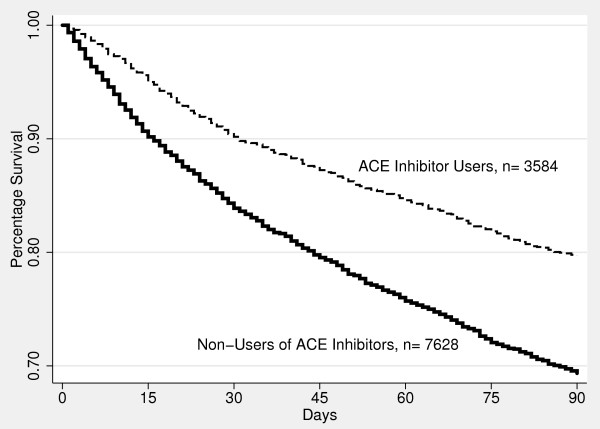
**Proportion of surviving patients hospitalized with COPD exacerbation by use of ACE inhibitor versus non-use (p < 0.001)**.

Figures 1 and 2 were created using Cox proportional hazard models to estimate, and graph, the baseline survivor functions for statin use (Figure 1) and ACE inhibitor use (Figure 2) over the first 90-days after admission. After adjusting for potential confounders, both statin and ACE inhibitor use were significantly associated with improved 90-day survival (p < 0.0001).

In the multilevel models, after adjusting for the appropriate propensity score and admitting hospital, prior use of a statin (odds ratio (OR) 0.51, 95% confidence interval (CI) 0.40–0.64) and ACE inhibitor use (OR 0.55, 95% CI 0.45–0.66) were significantly associated with decreased 90-day mortality. For the outcome of 30-day mortality we had similar findings with both statin (OR 0.51, 95% CI 0.41–0.64) and ACE inhibitor use (OR 0.58 95% CI, 0.48–0.70) associated with decreased 30-day mortality. In addition, to examine whether there were significant interactions between statins and ACE inhibitors use, we re-ran our model with medication variables and the individual potential confounders, rather than the propensity score. Our results were similar with both prior use of a statin (OR 0.47, 95% CI 0.37–0.60) and prior use of an ACE inhibitor (OR 0.59, 95% CI 0.51–0.69) significantly associated with decreased 90-day mortality, and there was no significant interaction between the ACE inhibitor and statin use.

In addition, to examine whether use of both medication classes may be associated with improved survival beyond that observed in subjects using statins or ACE inhibitors alone, we re-ran our model with 3 medication variables (statin use only, ACE inhibitor use only, or use of both statins and ACE inhibitors) and the individual potential confounders, rather than the propensity scores. We found that all 3 groups were associated with decreased mortality: ACE inhibitors alone (OR 0.62, 95% CI 0.53–0.73), statins alone (OR 0.49, 95% CI 0.39–0.61), and those using both (OR 0.40, 95% CI 0.32–0.52).

To further examine whether these associations were only for pre-existing comorbid conditions for which statins and ACE inhibitors have been shown to be protective (coronary artery disease, strokes, and diabetes) or potentially protective (congestive heart failure) we repeated our primary multivariable analyses stratified by each of these conditions. Table 3 shows these results. Except for ACE inhibitors in subjects with a history of stroke, all other odds ratios and confidence intervals were statistically significant and similar to the analyses including the entire population.

**Table 3:** Results of multilevel models by history of specific comorbidities

	**History of comorbid condition**	
	Yes	No
Myocardial infarction		
ACE inhibitor	0.60 (0.43–0.85)	0.63 (0.54–0.74)
Statin	0.39 (0.27–0.58)	0.53 (0.44–0.65)
Congestive heart failure		
ACE inhibitor	0.61 (0.51–0.74)	0.59 (0.47–0.73)
Statin	0.63 (0.51–0.80)	0.37 (0.29–0.49)
Stroke		
ACE inhibitor	0.75 (0.54–1.05)	0.61 (0.52–0.71)
Statin	0.41 (0.27–0.63)	0.54 (0.44–0.65)
Diabetes		
ACE inhibitor	0.62 (0.47–0.80)	0.66 (0.56–0.78)
Statin	0.49 (0.36–0.67)	0.54 (0.44–0.66)

* Results reported as odds ratio (95% confidence interval)

In the multilevel model that examined non-statin lipid lowering medication use there was no significant association with mortality (OR 0.98, 95% CI 0.62–1.54).

## Discussion

We found that prior outpatient use of statins and ACE inhibitor was associated with decreased 90-day mortality for subjects ≥ 65 years of age hospitalized with acute COPD exacerbations. Further studies, including randomized control trials, are needed to examine the impact of statins and ACE inhibitors, both pre-hospitalization and acute, for subjects who are at risk for acute exacerbation of COPD and COPD-related mortality.

Our study supports the recent findings that subjects with COPD, or hospitalized with acute exacerbation of COPD who were on statins and/or ACE inhibitors at admission had significantly improved outcomes [16-19]. Unfortunately those studies were limited by having small sample sizes at a single site [18,19], only examining in-hospital mortality [16], and not adjusting for other major comorbidities that may be associated with clinical outcomes [17]. In addition, only one prior study reported the impact of ACE inhibitor use on COPD-related mortality [17].

Soyseth and colleagues [18] examined 854 patients who were hospitalized with acute exacerbation of COPD from a single Norwegian hospital, and followed them for a median of 1.9 years. They found that statins (hazard ratio (HR) 0.57, 95% CI 0.38–0.87) and statins along with inhaled corticosteroids (HR 0.39, 95% CI 0.22–0.67) were associated with decreased long-term mortality.

Frost and colleagues published a paper in Chest [16], which described a cohort study and 2 small case-control studies (397 patients with influenza and 207 patients with COPD-related deaths) to examine the association of statin therapy with mortality. They found that for those subjects who received > 4 mg/day of statins for at least 90-days prior to death had significantly lower odds of death from influenza/pneumonia (OR 0.60, 95% CI 0.44–0.81) and COPD (OR 0.17, 95% CI 0.07–0.42). Unfortunately this study was limited by including only examining in-hospital mortality and using discharge diagnosis as the cause of death, problems with the exposure definition [32], not adjusting for steroid use, and not using techniques to minimize immortal time bias [33], which is defined as a span of follow-up during which, because of exposure definition, the outcome under study could not occur [34].

Mancini and colleagues [17] conducted a nested case-control study of subjects with COPD from 2 different populations: one of which only included patients who had recently undergone coronary revascularization and a second with no history of myocardial infarction but newly started on nonsteroidal anti-inflammatory drugs. Their multivariable models adjusted for other cardiac medications, pulmonary medications, and previous hospitalization for congestive heart failure and pneumonia. Mancini's findings suggested that statin use was associated with lower rates of hospitalization for COPD, myocardial infarction (MI), and all-cause mortality for both low- and high-risk cardiovascular groups. ACE inhibitors and ARBs were associated with lower mortality in both groups but had variable associations for MI and no significant association with hospitalization for COPD. However, the researchers failed to adjust for major comorbid conditions that are associated with mortality and hospitalization such as a prior stroke, diabetes, peripheral vascular disease, etc., in their multivariable models [24,35]. In addition, they did not provide the number of subjects exposed to the medications of interests in the various case-control groups. In contrast to Soyseth's [18] paper they found no significant impact of steroid use upon the clinical outcomes examined, but they examined both oral and inhaled corticosteroid use together in their models rather than separately.

Although our study was a large database analysis and subject to the recognized limitations of such studies, we carefully assembled our cohort from complete patient discharge data to avoid ascertainment bias. Our sample was predominantly men due to our use of VA administrative data, and it is possible that women may have differential responsiveness to these medications as compared to men. Unfortunately due to the lack of pulmonary function data in these databases we had to rely upon ICD-9 codes and medication use to define COPD. However, a recent publication by Joo et al. demonstrated that using a similar methodology that > 90% of subjects classified as having COPD were GOLD class 3–4 [21]. In addition, prior studies have demonstrated that clinicians frequently treat patients without this data [36], and we have no reason to believe that there would be differences between those exposed to the medications of interest versus the non-exposed. Also as this was an administrative database study we were unable to verify the diagnosis of COPD exacerbation. However a similar definition has been previously used for another study of acute exacerbations of COPD [37]. Also, we are unable to assess factors such as inpatient management of the COPD exacerbation, inpatient continuation of the statins/ACE inhibitors or the dose effect due to the lack of availability of these data. Further research is needed to examine these factors. Finally, as in any non-experimental study, we are unable to state conclusively that the prior outpatient use of statins and/or ACE inhibitors is the cause of decreased mortality, especially since we are unable to examine causes of death. However, since subjects on these medications have numerous medical conditions that are significantly associated with increased short-term mortality, and our analyses were adjusted for several factors that are associate with patient frailty or "healthy user bias", we feel that we have good evidence that these medications may have beneficial effects for subjects hospitalized with acute COPD exacerbations.

A strength of our study is that our cohort has the same access to medical care, and low to no-cost prescriptions due to the structure of the VA health care system [38]. In addition, we adjusted for important sociodemographic characteristics and comorbid conditions in our models. Future observational studies need to adjust for these, and other potential characteristics, that may impact the prescription and use of these medications. Finally, our definition of medication exposure and follow-up minimized potential immortal time bias.

## Conclusion

In conclusion, our study finds that prior outpatient use of statins and ACE inhibitors was associated with decreased mortality for subjects hospitalized with acute COPD exacerbations. These results add an additional potential benefit of statin and ACE inhibitor use to the already compelling data for their use in subjects with vascular disease and diabetes. Randomized trials are needed to confirm the magnitude of the impact of statin and ACE inhibitor use, either pre-hospitalization or acute, on subjects hospitalized with acute exacerbation of COPD and to elucidate the mechanism(s) by which they may work.

## Abbreviations

ACE: Angiotensin converting enzyme; ARB: Angiotensin II receptor blocker; CI: Confidence interval; COPD: Chronic obstructive pulmonary disease; FY: Fiscal year; HR: Hazards ratio; MI: Myocardial infarction; OR: Odds ratio; VA: Department of Veterans Affairs.

## Competing interests

None of the authors, except for AA and MIR, have any conflicts of interests to disclose regarding this paper. AA has served on the speaker's bureaus of Pfizer, Boehringer Ingelheim, GlaxoSmithKline, Astra-Zeneca; Ortho-McNeil-Janssen, Johnson & Johnson; Bayer-Schering Pharma, Schering- Plough; in the advisory board of GlaxoSmithKline, Boehringer Ingelheim, Bayer-Schering Pharma, Schering- Plough, and Pfizer; and is the principal investigator on research grants that were awarded to the University of Texas Health Science Center at San Antonio by GlaxoSmithKline, Pfizer, and Lilly Pharma. MIR is a consultant for Ortho-McNeil-Janssen, Johnson & Johnson, and Pfizer; and on the speaker's bureau for Ortho-McNeil-Janssen, Johnson & Johnson, GlaxoSmithKline, Pfizer, Covidien and BARD, Inc.

## Authors' contributions

EMM originated and designed the study, was primarily responsible for analysis of the data, and preparation of the paper.

LAC contributed to the design of the study, contributed to the analysis of the data, and preparation of the paper.

MJP contributed to the design of the study, contributed to the analysis of the data, and preparation of the paper.

MIR contributed to the design of the study and preparation of the paper.

RMM contributed to the design of the study and preparation of the paper.

BN contributed to the design of the study, contributed to the analysis of the data, and preparation of the paper.

AA originated and designed the study, contributed to the analysis of the data, and preparation of the paper.

All authors approved the final manuscript.
